# Detection and molecular characterization of *Avipoxvirus* in *Culex* spp. (Culicidae) captured in domestic areas in Rio de Janeiro, Brazil

**DOI:** 10.1038/s41598-022-17745-4

**Published:** 2022-08-05

**Authors:** Carolina Soares van der Meer, Patrícia Gonzaga Paulino, Talys Henrique Assumpção Jardim, Nathália Alves Senne, Thamires Rezende Araujo, Daniele dos Santos Juliano, Carlos Luiz Massard, Maristela Peckle Peixoto, Isabele da Costa Angelo, Huarrisson Azevedo Santos

**Affiliations:** 1 Departamento de Epidemiologia e Saude Pública, Instituto de Veterinaria, Universidade Federal Rural do Rio de Janeiro, Rodovia BR 465, Km 07, Seropedica, RJ 23890-000 Brazil; 2 Departamento de Parasitologia Animal, Instituto de Veterinaria, Universidade Federal Rural do Rio de Janeiro, Rodovia BR 465, Km 07, Seropedica, RJ 23890-000 Brazil

**Keywords:** Environmental microbiology, Parasitology, Virology

## Abstract

Avian pox is a highly contagious poultry disease that causes significant economic losses. Mosquitoes belonging to the genus *Culex* (Diptera: Culicidae) have a fundamental role in disseminating *Avipoxvirus* (Poxviridae). This study proposes investigating the presence of *Avipoxvirus* (APV) DNA in *Culex* spp. from Rio de Janeiro to determine its frequency and perform a phylogenetic analysis based on the core like the 4b protein (*p4b)* gene. The detection of APVs was conducted individually on four hundred *Culex* spp. mosquitoes. A total of 12.23% (47/384) of the *Culex* spp. were positive in the PCR. Sequencing the *p4b* gene revealed that this study’s sequences displayed 98.8–99% identity with *Fowlpoxvirus* (FWPW) sequences available in GenBank. In the phylogenetic analysis, these APVs were clustered in the A1 subclade together with FWPW sequences from several countries. The evolutionary distance of the *p4b* gene was 0.61 ± 0.21% in rural areas and 0.38 ± 0.16% in peri-urban areas. The current investigation is the first study to report the detection of APVs in field-caught mosquitoes. Moreover, a high frequency of APV DNA was observed in *Culex* spp. captured in domestic areas, where backyard poultry is present. This data demonstrates the importance of implementing control measures for *Culex* spp. to mitigate the transmission of APVs in backyard poultry in Rio de Janeiro.

## Introduction

Avian pox is an important viral disease with a high incidence in tropical and subtropical countries^1^. Caused by a double-stranded DNA virus of the genera *Avipoxvirus* from the Poxviridae family, which infects and produces clinical signs in numerous domestic and wild birds^2,3^. Avipoxviruses (APVs) have a cosmopolitan distribution and can affect any avian species, with no predilection for gender or age, despite being more common and deadly in young birds^4,5^. In domestic avian species, particularly in commercial poultry production, APVs have a relevant health impact^6^. Furthermore, APVs can also have relevant impacts on the health of wild bird species. In some cases, APV infections put the conservation of the affected avian species at risk, especially in outbreaks where the virus is introduced in non-adapted ecosystems^7–9^.

The means of transmission includes arthropods (such as biting midges, flies, mosquitoes, and mites) acting as mechanical vectors^5,10–12^. APV propagation can also be carried out by aerosols generated by infected birds, direct contact with injuries, and the ingestion of contaminated food or water^13^. Previous studies have reported the vital role of the cosmopolitan species *Culex quinquefasciatus* (Say, 1823) and *Aedes aegypti* (Linnaeus, 1762) as mechanical vectors in APV transmission^5,14^, which takes place due to the permanence of viable viral particles in the mosquitoes’ proboscis, remaining for up to 14 days^15^. Furthermore, global warming could increase arthropod-borne diseases, both in humans and wildlife^16,17^. This tendency might be even worse in regions suffering from a history of deforestation, such as the Southeastern region of Brazil^18,19^.

APV diversity is primarily classified into three phylogenetical groups (A, B, C), characterized by Fowlpoxvirus (FWPW) (clade A; subdivisions A1–A7), Canarypoxvirus (clade B; subdivisions B1-B3), and Psittacinepoxvirus (clade C)^20^. However, two other clades (D, E) have been proposed^21,22^. Clade D includes a unique APV strain, QP-241, isolated from a Japanese quail collected in Italy^21^. The sequences included in clade E were earlier recorded from outbreaks in turkey herds in Hungary^22^ and layer chickens in Mozambique^23^. Ribeiro et al.^24^ also reported fowls from the Southern region of Brazil presenting APV sequences clustering in clade E. However, no investigation as to the viral agent in mosquitoes has been performed in Brazil. Moreover, few studies have targeted the circulation of APVs and their identification in mosquito species with vectoring capacities, such as mosquitoes of the *Culex* genus. Furthermore, it presents an analysis of the phylogenetic relationship and the genetic variability of APVs from *Culex* spp. collected in rural and peri-urban areas nearby backyard poultry located in the municipality Seropedica, Rio de Janeiro, Brazil.

## Results

**Figure 1:**
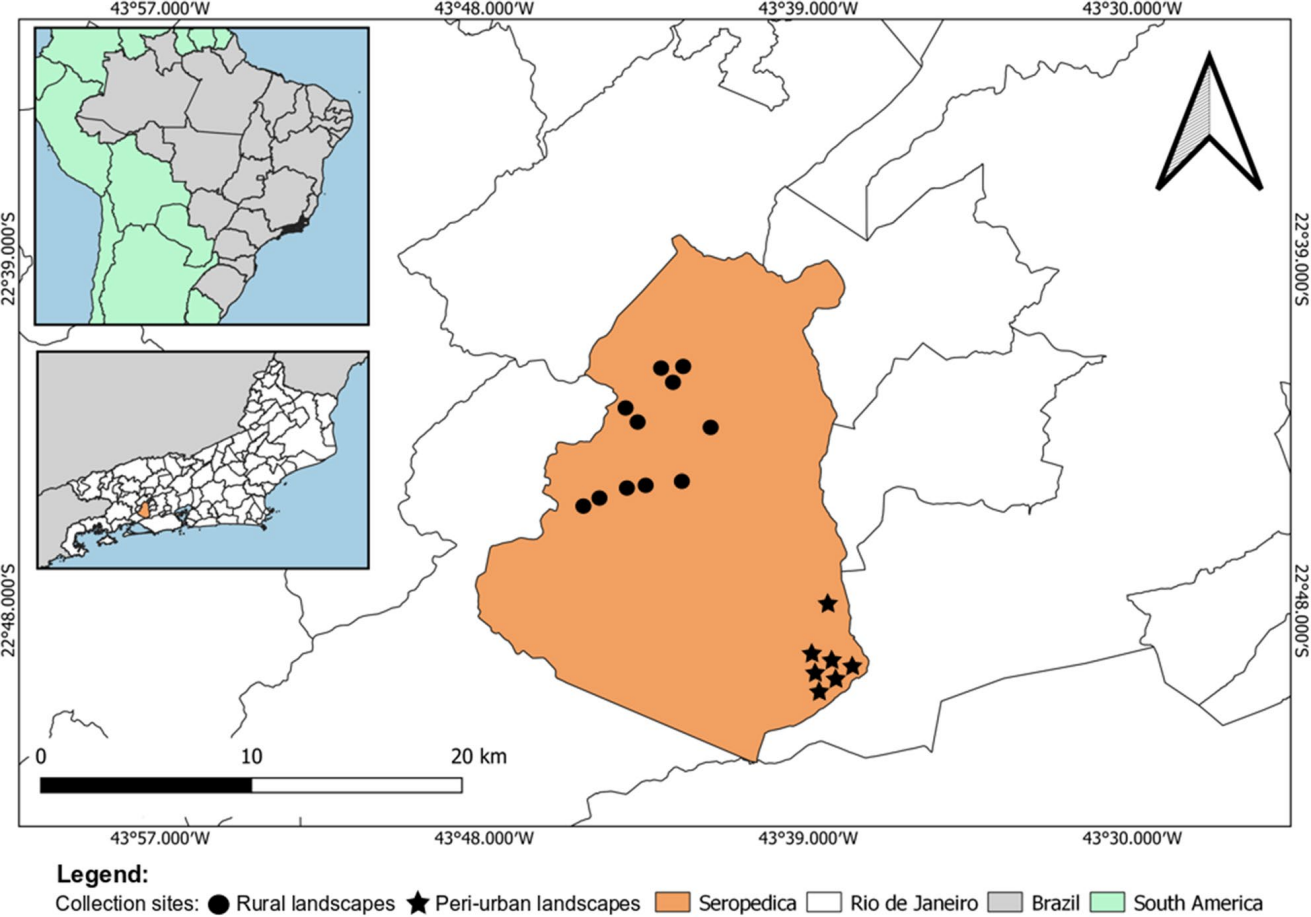
The geographic location of Seropedica, Rio de Janeiro, highlighting the capture points of *Culex* mosquitoes in subsistence breeding of *Gallus gallus* located in rural and peri-urban areas. Map created in QGIS software 3.22.8 'Białowieża' (https://qgis.org/pt_BR/site/).

**Figure 2:**
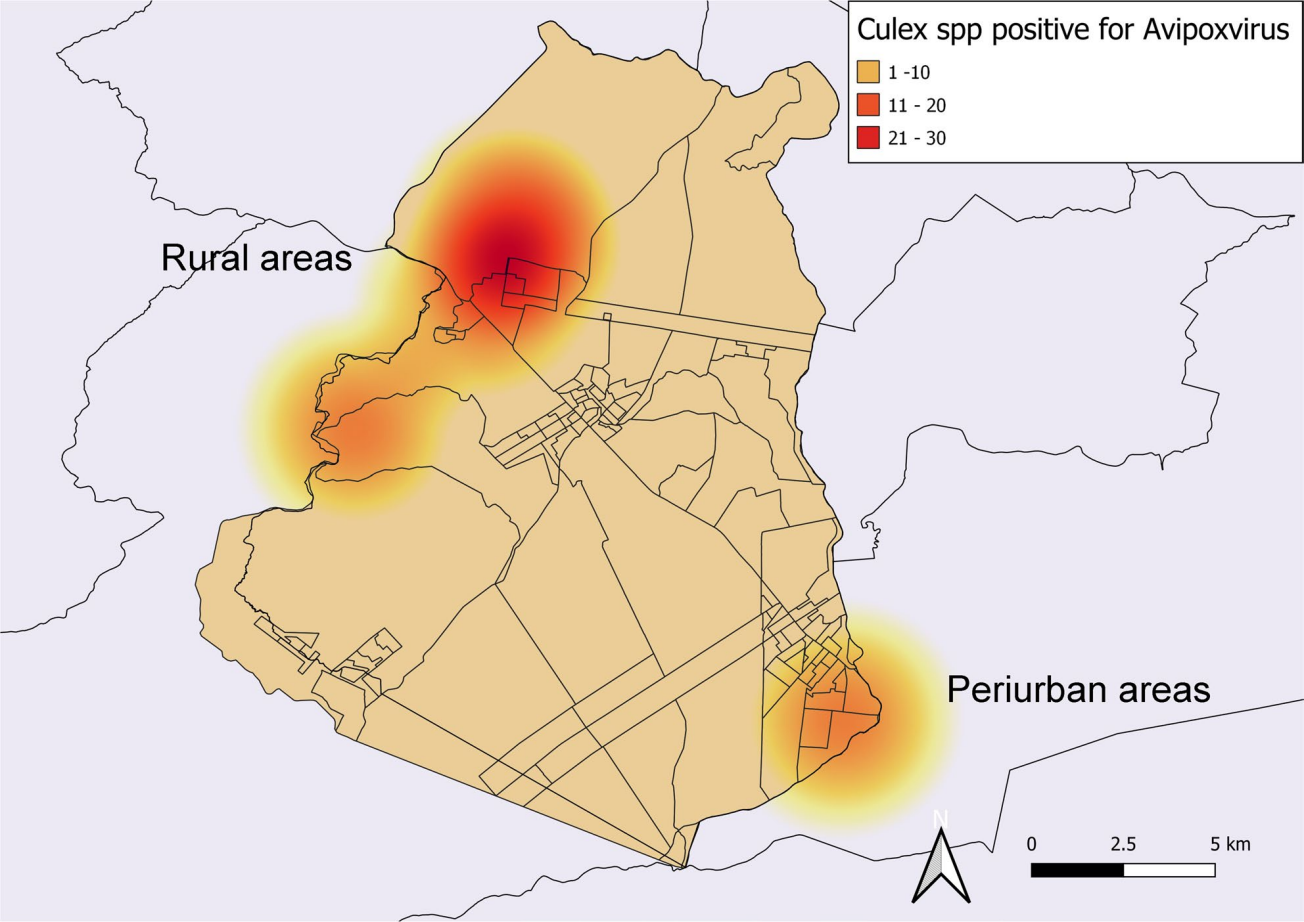
Kernel map showing the concentration of *Culex* spp. positive for *Avipoxvirus* in rural and peri-urban areas in Seropedica, RJ. Map created in QGIS software 3.22.8 'Białowieża' (https://qgis.org/pt_BR/site/).

The PCR applied in the current study, which targets APVs, presented a detection limit of 100 copies of the APV *p4b* gene fragment. The molecular screening resulted in a frequency of 12.23% (47/384) in *Culex* mosquitoes. Of these positive samples, 13.63% (21/154) were from peri-urban areas, and 11.30% (26/230) were from rural areas (Fig. 1). There was no significant statistical association between the frequency of APVs in *Culex* mosquitoes and the analyzed areas (p > 0.05). However, the kernel map identified a hot zone, demonstrating a higher concentration of APV-positive mosquitoes in rural areas of the municipality of Seropedica (Fig. 2).

The qPCR targeting the *chicken mitochondrial* gene revealed that 91.49% (43/47) of the *p4b*-positive APV samples were also positive for chicken blood, and 8.51% (4/47) of the *p4b*-positive APV samples were negative for chicken blood.

**Figure 3:**
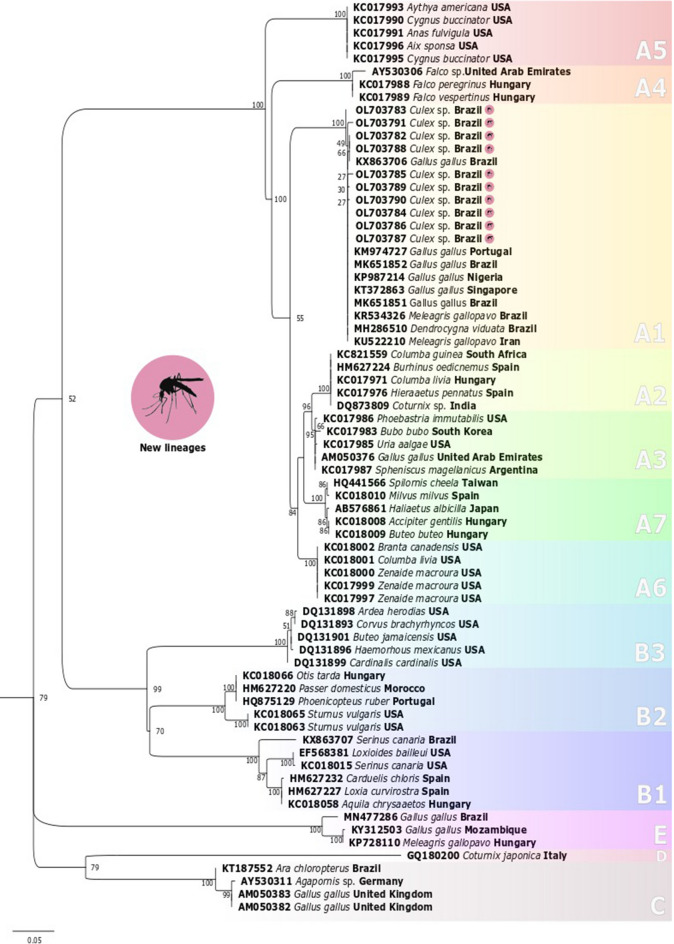
Phylogenetic tree estimated by the maximum likelihood method from partial *p4b* gene sequences of *Avipoxvirus* isolated in this study (highlighted) compared to sequences available in GenBank. The numbers on the branches indicate the bootstrap value out of 1000 replicates. The bar represents five substitutions per 100 nucleotide positions.

A total of ten positive samples were sequenced for phylogenetic reconstruction based on the *pb4* gene. The identity percentage of the sequences in this study ranged from 98.8 to 99%, with FWPW sequences available in the GenBank database. All sequences recovered from *Culex* mosquitoes in this study were clustered within clade A, subclade A1, and denoted as part of the FWPW category. In the same group, the A1 subclade isolates were recovered in *Gallus gallus* from Brazil, Nigeria, Portugal, and Singapore; *Meleagris gallopavo* from Iran and Brazil; and *Dendrocygna viduata* from Brazil (Fig. 3). The global evolutionary distance of the FWPW *p4b* gene was 0.48 ± 0.14% in the targeted region. The evolutionary distance between FWPW sequences from mosquitoes collected in rural and peri-urban areas was 0.47 ± 0.13%. The evolutionary distance was compared within each evaluated area, obtaining a value of 0.61 ± 0.21% in the rural area and 0.38 ± 0.16% in the peri-urban area (Table 1).

**Table 1:** Evolutionary distance (%) between *p4b* gene sequences of *Avipoxvirus* obtained from *Culex* spp. in rural and peri-urban areas in the municipality of Seropedica, Rio de Janeiro, Brazil.

	1	2	3	4	5	6	7	8	9
OL703782—Rural area									
OL703783—Rural area	0.38								
OL703784—Rural area	0.19	0.19							
OL703785—Peri-urban area	0.76	0.76	0.57						
OL703786—Peri-urban area	0.19	0.19	0.00	0.57					
OL703787—Peri-urban area	0.19	0.19	0.00	0.57	0.00				
OL703788—Peri-urban area	0.00	0.38	0.19	0.76	0.19	0.19			
OL703789—Peri-urban area	0.38	0.38	0.19	0.76	0.19	0.19	0.38		
OL703790—Rural area	0.38	0.38	0.19	0.76	0.19	0.19	0.38	0.38	
OL703791—Rural area	1.15	1.15	0.95	1.54	0.95	0.95	1.15	1.15	1.15

## Discussion

Brazil is the second leader in the world’s poultry production and the first leader in chicken exportation^25^. Therefore, any negative impact on poultry health can result in substantial commercial losses. In this context, it is of utmost interest to investigate all aspects of poultry health. Avian pox is a viral disease with a high incidence in tropical and subtropical countries^1^. Avipoxviruses have no predilections in terms of host species, sex, or age, targeting more than 374 domestic and wild bird species and making it difficult to achieve environmental eradication^3^. Furthermore, arthropods perform APV transmission, which further exacerbates the potential of dispersion. Mosquitoes are one of many arthropods playing the role of mechanical vector in the Avian pox epidemiological chain, which is a potent threat given that mosquitoes populations in the tropics are high^26^.

Considering the substantial economic loss caused by Avian pox, it is vital to elucidate the transmission chain of APVs by identifying potential transmission sources^5^. Although investigations targeting APVs in mosquitoes are scarce, prior studies have demonstrated that APV detection in potential vectors is possible and can become an essential tool for monitoring the pathogen circulation in areas where the disease occurrence may increase due to climatic factors. In addition, APV detection in potential vectors can further clarify the mosquitoes’ participation in the APV transmission chain in a determined area, which could improve the strategic measures to control the Avian pox disease. Furthermore, the present study can contribute to the clarification of the biology of the vectors involved in and strategic measures for vector control, which is of veterinary interest^5,27^.

The frequency of APVs found in field-caught *Culex* spp. collected in the present investigation (12.23%; 47/384) was higher than previously recorded by Yeo et al.^5^, who observed a positivity of 2.60% (4/154) in mosquito pools collected in areas where outbreaks of Avian pox occurred in Singapore. This divergence may be related to several factors, such as differences in the sampling approaches adopted in these studies, sample conservancy, and the chosen collection sites. Although the studies by Yeo et al.^5^ and Lee et al.^12^ chose the PCR method for the detection of *Avipoxvirus* in mosquito and biting midge pools, respectively, the present study applied a distinct sampling method by individually testing *Culex* specimens. Individual sampling proved to be sufficient to obtain the total amount of DNA after performing the modified extraction technique outlined by Ayres et al.^28^. This was demonstrated by quantification through spectrophotometry and by checking the quality of genomic DNA targeting the mitochondrial *cytochrome oxidase subunit I* (*COI*) gene, which was of good quality at the expected height^28^. Moreover, the DNA extracted from the samples obtained in the current investigation was preserved in a DNA stabilization solution and maintained in an ultra-freezer at − 80 °C until molecular detection.

Notably, the selected spots were located in areas where outbreaks of Avian pox had occurred in previous years. However, during the present investigation, no Avian pox case was observed or recorded close to the studied points by the owners or local veterinarians. Despite the absence of the disease’s clinical signs in backyard chickens, the frequency of APVs in *Culex* spp. was relatively high in the target area, raising questions about the involvement of other domestic birds species participating in the maintenance of virus circulation. The presence of four *Culex* spp. that were positive for APVs and negative for the *chicken mitochondrial* gene reinforced this hypothesis. Therefore, more studies must be performed to elucidate the transmission chain of APVs in the targeted area.

Prior studies targeting APVs-*p4b* obtained of avian species from Brazil demonstrate the presence of several APVs lineages of distinct clades circulating throughout the country. Studies conducted in the Southeastern region of Brazil, the same area of the current research, have reported the presence of APV sequences clustered in subclade A1 and isolated from both turkeys and chickens^29,30^. Moreover, in the Northeastern region of Brazil, Braga et al.^31^ isolated APVs with lineage clustering in the A1 subclade coming from a Brazilian native duck (*Dendrocygna viduata*) (Fig. 3). The present phylogenetic reconstruction exposes the lineages of APVs recovered from *Culex* mosquitoes from the state of Rio de Janeiro, also grouped in the A1 subclade (APVs-A1) (Fig. 3).

Historically, the lineages of APVs from subclade A1 were strongly associated with Galliform hosts (*Gallus gallus* and *Meleagris gallopavo*) according to the results published by Gyuranecz et al.^20^, Jarmin et al.^32^, and Manarolla et al.^21^. Nevertheless, recent records of the APVs-A1 sequence infecting a Brazilian native duck (Order Anseriformes) reported by Braga et al.^31^ have raised questions about this subclade’s specificity within the species of the Galliformes order. Furthermore, given the fact that the most common APV host species of subclade A1 (*Gallus* and *Meleagris gallopavo*) are exotic species introduced by humans as food resources in most regions of the globe, such as in Brazil, it is possible to suggest that the lineages of APVs of the A1 subclade accompany their hosts in species introduction around the globe. However, given the record of an APVs-A1 infecting a native Brazilian species^31^, it is possible to suggest a community APVs-A1 transmission between different avian host species.

The species-level identification in *Culex* spp. requires genitalia dissection, making such samples unviable for molecular analysis. *Cytochrome oxidase I* (*COI*) DNA barcoding contains information for identifying mosquitoes of the *Culex* genus^33^. However, a criticism of using the COI barcode for specification is the ambiguous identification or the absence of clusters in phylogenetic trees of recently diverged species^34–36^. Algorithms were developed by Meier et al.^37^ and van Velzen et al.^38^ to improve COI barcoding identification at the species level. However, Laurito et al.^39^ employed a COI barcode and BCM algorithm to identify *Culex* spp. from Brazil and Argentina and concluded that the COI barcode does not contain enough information to distinguish Culex spp. Thus, the present study could not determine the real prevalence of APVs associated with a particular *Culex* species. Nevertheless, most *Culex* species found in peri-urban and rural areas in the state of Rio de Janeiro are highly ecologically similar^40–45^. Therefore, the differences do not substantially impact the control measures applied to *Culex* species and the transmission of APVs.

The present research reports on the genetic diversity of APVs. The most divergent APV sequences were obtained in mosquito samples from rural areas. This result may indicate pathogen adaptation to highly anthropized areas since significant genetic divergence is present in areas with lower anthropization levels due to more significant interactions and an abundance of host species found in these regions. Giraudeau et al.^46^ demonstrated for the first time that the highest rates of *Avipoxvirus* infection in finches (*Haemorhous mexicanus*) were in urban areas, where human activities were more often present.

## Methods

### Study area

The present study was conducted in the municipality of Seropedica (22° 44′ 38″ south latitude; 43° 42′ 27″ west longitude) in Rio de Janeiro as represented in the Fig. 1, from June 2016 to July 2017. Mosquito collections were carried out in properties with backyard poultry from rural and peri-urban areas.

### Mosquito collections and identification of *Culex* spp

The parameters of an infinite population were considered based on the desired level of prevalence to determine the sample size^47^, with a sample error of 5%, a confidence level of 95%, and an expected prevalence of 50%, resulting in a total of 384 mosquitoes in sampling. The collections were carried out using CDC light traps in peri-domestic areas, which operated for 12 h, three times a week, totaling 864 h of operation of the traps from July 2016 to July 2017. A total of 2839 mosquitoes of the *Anopheles*, *Aedes*, and *Culex* genera were collected, with the *Culex* genus being the most abundant and comprising 96.23% (2732/2839). The identification of mosquitoes at the genus level was performed by morphological structures common to the *Culex* spp. according to Forattini^43^ and Berlin and Belkin^48^. Characteristics such as sex and engorgement were considered. The specimens were individually placed in tubes containing RNAlater® solution and stored at – 20 °C until molecular analysis was performed.

### DNA extraction

DNA extraction was performed for each specimen of *Culex* spp. Before the extraction protocol, each specimen stored in RNAlater® (ThermoFisher Scientific) had its eyes dissected and discarded to avoid possible inhibitory action in molecular analysis caused by pigmentary components from insect eyes^49^. Each body was washed three times with 500 μL of sterile PBS and centrifuged at 14,500×*g* for 3 min to remove the RNAlater® solution. A protocol published by Ayres et al.^28^ was performed for DNA extraction. All samples were quantified by Nanodrop® ND-2000 spectrophotometry (Nanodrop Technologies, DE, USA), and samples were standardized at 30 ng/μL^28^.

### Amplification of cytochrome C oxidase subunit I (COI) gene in *Culex* spp. DNA

A polymerase chain reaction (PCR) based on the mitochondrial COI gene, considered barcoding for mosquito identification^33^, was performed to verify DNA quality. The assay was performed using the universal primers LCO1490 (5'-GGTCAACAAATCATAAAGATATTGG-3') and HCO2198 (5'-TAAACTTCAGGGTGACCAAAAAATCA-3'). A final volume of 12.5 μL, containing 1 × PCR buffer, 2.5 mM of MgCl2, 0.2 mM of each dNTP, 0.5 mM of each primer, 1 U of Platinum Taq DNA Polymerase (Invitrogen®), and 3 μL of *Culex* spp. DNA. The thermocycling conditions were as follows: 94 °C for 3 min, followed by 35 cycles of 94 °C for 30 s, 60 °C for 30 s, 72 °C for 45 s, and a final extension at 72 °C for 10 min on the ProFlex™ 3 × 32-well PCR System (ThermoFisher Scientific). The amplified products were subjected to electrophoresis on a 1.5% agarose gel in a 1 × TAE Buffer. Then, the gels were stained by immersion in ethidium bromide (5 mg/μL) and visualized under ultraviolet light on UV Transilluminator, E-gel electrophoresis system (Invitrogen, ThermoFisher Scientific).

### PCR assay targeting *p4b* gene of *Avipoxvirus* in *Culex* spp

A pair of primers was selected to detect the *Avipoxvirus* based on the *p4b* gene, which encodes the viral nucleocapsid protein. These primers are widely used for the molecular detection of *Avipoxvirus* amplifying a PCR fragment of 578 bp^50,51^. The positive control was obtained from the total DNA extracted from the lyophilized vaccine Bouba Aviária Suave Biovet®. The vaccine content aliquots were resuspended in 1 × sterile phosphate saline buffer (1 × PBS) with pH 7.2 and extracted using the Invitrogen™ PureLink™ Genomic DNA Mini Kit. All extracted aliquots were quantified by Nanodrop® ND-2000 spectrophotometry (Nanodrop Technologies), standardized at a concentration of 30 ng/μL of total DNA for molecular analysis.

The PCR assay was optimized to achieve the greatest analytical sensitivity. All samples were submitted for *Avipoxvirus* detection assay on the ProFlex™ 3 × 32-well PCR System (ThermoFisher Scientific), with a final volume of 12 μL containing the following: 1 × PCR buffer (10 mM Tris–HCl; pH = 8.3; 50 mM KCl) (Invitrogen®), 1.5 mM Magnesium Chloride (MgCl2 50 mM, Invitrogen®), 0.2 mM of each nucleotide (dATP, dGTP, dTTP, and dCTP-100 mM Invitrogen®), 0.4 mM of primers, 1.2 U of Platinum Taq DNA Polymerase (Invitrogen®) and 1.5 µL of total DNA at 30 ng/µL. The thermocycling conditions were 94 °C for 2 min; 35 cycles of 94 °C for 60 s; 60 °C for 30 s; 72 °C for 60 s; and a final extension at 72 °C for 2 min. The amplified products were submitted to electrophoresis on a 2.0% agarose gel in a 1 × TAE buffer. The PCR products were purified using the Wizard® SV Gel Kit and PCR Clean-Up System (Promega™, Madison, WI, USA) following the manufacturer’s recommendations for sequencing.

### Analytical sensitivity of *Avipoxvirus-p4b*-PCR

The analytical sensitivity of the PCR was assessed through the limit of detection (LOD), which was determined according to serial decimal dilutions carried out in triplicate of the p4b-PCR amplicon. The PCR amplicon was purified and quantified by the Qubit fluorometer (ThermoFisher Scientific). This amplicon concentration was employed to calculate the copy number using the following equation: copy number = (6.02 × 10^23^ [copies per mole] × p4b-PCR amplicon concentration [g])/(578 bp [target size] × 660 [g/mol/bp]). The number of copies ranged from 10^6^ (one million) to 10^–1^ (zero) in eight different dilutions performed for further evaluation.

### qPCR assay targeting chicken mitochondrial gene

The qPCR assay employed the primers targeting the chicken mitochondrial gene designed by Lahiff et al.^52^ and was performed with 1 × Meltdoctor buffer, 0.6 uM of each primer, and 1.5 µL of total DNA at 30 ng/µL. The thermocycling conditions were as follows: 95 °C for 10 min, 95 °C for 15 s, 57 °C for 30 s, 72 °C for 30 s for 40 cycles, and 72 °C for 10 min.

### Sequencing and phylogenetic analysis of *Avipoxvirus*

The sequencing of positive samples for *Avipoxvirus* was performed using the Applied Biosystems 3730XL DNA Sequencer. The quality of the sequences was analyzed using the CLC Main Workbench Version 7.2 software (Qiagen®, Hilden, Germany) through the evaluation of the chromatogram. The contigs were assembled in the same software, and the similarity of each sequence was obtained using the BLASTn tool available at https://blast.ncbi.nlm.nih.gov/. The phylogenetic reconstruction was performed with 61 sequences available in GenBank belonging to subclades A1 to A7, B1 to B3, clades C, D, and E were compared to the samples obtained in this study. Sequences were aligned in MAFFT software using default options, and then the matrix was visually inspected for inconsistencies^53^. After removing misaligned positions with GBlocks, according to Talavera and Castresana^54^, a final matrix was obtained. The best replacement model was determined using JModelTest software^55^. The inference of the *Avipoxvirus* phylogeny was performed using the Maximum Likelihood (ML) method. The clade bootstrap values were evaluated using the RaxML self-convergence criterion with the best pseudo-replica values^56^.

### Georeferencing

The Brazilian state cartographic base was collected in shapefile format from the electronic database of the Brazilian Institute of Geography and Statistics^57^ and uploaded in the GIS software [QGIS 3.22.8 'Białowieża' (https://qgis.org/pt_BR/site/], maintaining the coordinates in degrees in the Geocentric Reference System for the Americas (SIRGAS 2000, previously established by IBGE.

Thematic maps were created by applying the geographical coordinates of the collected samples in the cartographic base. A kernel map was also designed to visually distinguish infection clusters through interpolating the *Avipoxvirus*-positive samples and the study sites. The colored scale in the obtained raster was proportional to the frequency, ranging from 1 to 9.

### Statistical analysis

The frequency of *Avipoxvirus* in *Culex* spp. captured in the municipality of Seropedica in the state of Rio de Janeiro was compared using the Chi-Square (χ^2^) test, admitting an error of 5% using R software version 3.6.1^58^.

## Conclusion

This study reported a high frequency of FWPW in *Culex* spp. in Seropedica, indicating a risk in implementing an organic system for poultry production in Seropedica, Rio de Janeiro. The FWPW circulation in this area reinforces the importance of vaccination as a preventive measure. In addition, this is the first study to report FWPW sequences obtained from *Culex* mosquitoes collected in the southwestern region of Rio de Janeiro, Brazil.

## Data Availability

The dataset generated and analyzed during the current study is available in the GenBank repository. The sequences are deposited under the following accession numbers: OL703782, OL703783, OL703784, OL703785, OL703786, OL703787, OL703788, OL703789, OL703790, and OL703791. https://www.ncbi.nlm.nih.gov/popset/2271451140.
